# Administration of a Toll-Like Receptor 9 Agonist Decreases the Proviral Reservoir in Virologically Suppressed HIV-Infected Patients

**DOI:** 10.1371/journal.pone.0062074

**Published:** 2013-04-26

**Authors:** Anni A. Winckelmann, Lærke V. Munk-Petersen, Thomas A. Rasmussen, Jesper Melchjorsen, Thomas J. Hjelholt, David Montefiori, Lars Østergaard, Ole S. Søgaard, Martin Tolstrup

**Affiliations:** 1 Department of Infectious Diseases, Aarhus University Hospital, Aarhus, Denmark; 2 Duke University Medical Center, Durham, North Carolina, United States of America; University Hospital Zurich, Switzerland

## Abstract

Toll-like receptor (TLR) agonists can reactivate HIV from latently infected cells in vitro. We aimed to investigate the TLR-9 agonist, CPG 7909's *in vivo* effect on the proviral HIV reservoir and HIV-specific immunity. This was a post-hoc analysis of a double-blind randomized controlled vaccine trial. HIV-infected adults were randomized 1∶1 to receive pneumococcal vaccines with or without 1 mg CPG 7909 as adjuvant at 0, 3 and 9 months. In patients on suppressive antiretroviral therapy we quantified proviral DNA at 0, 3, 4, 9, and 10 months (31 subjects in the CPG group and 37 in the placebo-adjuvant group). Furthermore, we measured HIV-specific antibodies, characterized T cell phenotypes and HIV-specific T cell immunity. We observed a mean reduction in proviral DNA in the CPG group of 12.6% (95% CI: −23.6–0.0) following each immunization whereas proviral DNA in the placebo-adjuvant group remained largely unchanged (6.7% increase; 95% CI: −4.2–19.0 after each immunization, p = 0.02). Among participants with additional cryo-preserved PBMCs, HIV-specific CD8+ T cell immunity as indicated by increased expression of degranulation marker CD107a and macrophage inflammatory protein 1β (MIP1β) tended to be up-regulated following immunization with CPG 7909 compared with placebo as adjuvant. Further, increasing proportion of HIV-specific CD107a and MIP1β-expressing CD8+ T cells were strongly correlated with decreasing proviral load. No changes were observed in T cell phenotype distribution, HIV-specific CD4+ T cell immunity, or HIV-specific antibodies. TLR9-adjuvanted pneumococcal vaccination decreased proviral load. Reductions in proviral load correlated with increasing levels of HIV specific CD8+ T cells. Further investigation into the potential effect of TLR9 agonists on HIV latency is warranted.

## Introduction

Despite suppressive highly active antiretroviral therapy (HAART) human immunodeficiency virus (HIV) persists in all infected individuals as evidenced by the presence of low-level viremia [1], integrated proviral DNA [2] and rapid viral rebound following treatment interruption, which necessitate life-long therapy [3], [4].

Latently infected CD4+ T cells appear to be the primary barrier preventing eradication of the HIV infection by HAART [5]. This reservoir is established during primary HIV infection[6] either when newly infected CD4+ T cells revert to a silent memory state upon integration of HIV DNA into the host cell genome or when the virus directly infects a resting CD4+ T-cell. The transcriptional silence during resting cell infection enables viral evasion from immune-mediated clearance. However, replication competence is maintained and can be resumed upon subsequent activation of the cell [7]. While early initiation of HAART limits the size of the reservoir [8], [9], intensification of HAART appears to have limited effect on the proviral reservoir [10]–[13].

A large number of substances that reactivate HIV-1 expression in latently infected cells are currently investigated *in vitro* and *in vivo*, such as histone deacetylase inhibitors (HDACis)[14], IL-7[15], prostratin[16] and disulfiram[17], with the aim of finding new therapies that could eradicate the HIV-reservoir. To eradicate the latent HIV-reservoir, activation of latent virus must be followed by elimination of infected host cells by viral cytopathic effects, cytolytic T lymphocytes (CTLs) or antibody-mediated mechanisms. However, chronic HIV-infection is characterized by an impaired cytolytic capacity of CD8+ T cells, which is not restored with HAART [18]. A recent *in vitro* study showed that Vorinostat (an HDACi) increased virus production in latently infected resting CD4+ T cells, but did not lead to the removal of these cells[19]. Subsequent experiments showed that antigen-specific stimulation of patient CTLs led to efficient killing of reactivated cells emphasizing the need to consider enhancing HIV-specific immunity in eradication strategies [19].

Multiple therapeutic approaches have been tested to boost immunity against HIV in infected persons, both pathogen specific (e.g. therapeutic HIV/AIDS vaccines [20]–[22]) and non-pathogen specific (IL-2 and IL-7 administration [15], [23]). Non-pathogen specific stimulation of the innate immune system via toll-like receptors (TLRs) is used to treat certain viral diseases (e.g. Imiquimod, a TLR 7/8 agonist against genital warts) and as adjuvant in immunization (Cervarix, Heplisav, Ixiaro)[24]. Owing to their immune stimulatory properties via TLR9, synthetic CpG oligodeoxynucleotides (CpG ODNs) are used as vaccine adjuvants and have been shown to enhance vaccine immunogenicity in HIV-infected individuals and healthy adults with an acceptable safety profile [25]–[28]. Besides inducing humoral immunity, CPG-adjuvanted pneumococcal vaccine has been shown to enhance antibody-independent cellular immunity [29], [30]. In addition, CpG ODNs reactivate HIV-1 expression in latently infected cells by mechanisms that involve TLR9 signalling and eventually activation of NF-κB [31], [32], but the mechanistic details remain largely unresolved. Whereas increases in plasma HIV-RNA have been observed in HIV-infected patients with opportunistic bacterial infections[33], [34] and in HIV-infected patients treated with HIV-gag antisense ODN with CPG motif [35], it has not been investigated whether CpG ODNs decreases the latent viral reservoir *in vivo*.

Previously, we conducted a randomized controlled trial of safety and immunogenicity in which HIV-infected individuals were randomized to receive pneumococcal vaccination with or without CPG 7909 as a vaccine adjuvant [28]. An unexpected finding in this study was that 90.2% of the participants in the group receiving CPG as adjuvant experienced influenza-like symptoms after the third immunization compared to 4.3% in the placebo group. Given CpG's ability to reactivate HIV from latently infected cells *in vitro* [31], [32], we hypothesized that the observed systemic immunological events may have triggered the release of latent proviruses from long-lived cellular reservoirs. Hence, the CpG/pneumococcal vaccine trial provided a unique opportunity to evaluate the *in vivo* effect of a compound that in prior studies has been shown to enhance innate and cellular immunity[36] as well as to induce HIV-1 expression in latently infected cells [31], [32]. Therefore, we aimed to assess whether administration of CPG 7909 to HIV-infected individuals led to any changes in the size of the proviral reservoir. In addition, we evaluated the quantitative and qualitative effects of the CpG adjuvant on HIV-specific humoral and cellular immunity.

## Materials and Methods

### Study design

This is a post-hoc analysis of an investigator-initiated phase Ib/IIa, randomized, double-blind, placebo-controlled trial randomizing HIV-infected adults to immunization with pneumococcal vaccines with or without CPG 7909[28].

### Ethics statement

The study protocols were approved by the Danish Medicines Agency, the Regional Ethical Committee, and the Danish Data Protection Agency; and registered at www.clinicaltrials.gov (NCT00562939).

### Setting and participants

The study was conducted at the Department of Infectious Diseases, Aarhus University Hospital, Denmark. The study population included HIV-seropositive volunteers aged 18 or older. We excluded individuals who 1) had received PPV-23 immunization within the last 5 years; 2) were on antiretroviral therapy for less than 6 months; 3) were on antiretroviral therapy with HIV RNA >50 copies/mL; 4) with CD4+ cell count <200 cells/ µL; 5) were unavailable for first follow-up after first immunization. Written informed consent was obtained for all participants.

### Immunization and sample collection

Study details have been published elsewhere [28]. In brief, all participants were immunized with double the standard dose of PCV7 (Prevnar®, Wyeth) at 0 and 3 months and with one single dose of PPV-23 (Pneumo Novum®, Sanofi-Pasteur MSD) at 9 months. Participants were also seen at 4 and 10 months for immunogenicity and safety follow-up. One group received 1 mg CPG 7909 (Coley Pharmaceutical Group) formulated in 100 µL PBS buffer added to each of their three vaccine doses. The other group received PBS placebo buffer in place of CPG 7909.

### HIV DNA quantification

DNA was isolated from approximately 5×10^6^ (6×10^5^–9.6×10^6^) PBMCs using QIAmp® DNA blood midi kit according to the manufacturer's specifications (Qiagen, Denmark). The DNA was precipitated with ethanol and dissolved in TE-buffer to a final volume of 15 µL. Cell equivalents were based on RNAseP amplification. The following primers were used to target human RNAseP 5′-CCCCGTTCTCTGGGAACTC-3′ (forward) and 5′-TGTATGAGACCACTCTTTCCCATA-3′ (reverse) [37]. Amplification reactions were carried out in duplicates with a primer concentration of 0.5 µM, 10 µL SsoFast^TM^ EvaGreen® Supermix and 1 µL 100-fold diluted DNA as template in 20 µL total volume. Using the RNAseP genome copy number sample DNA was diluted to 100,000 cell equivalents per PCR reaction.

To determine the frequency of PBMCs carrying HIV DNA we prepared a standard curve obtained by serially diluted DNA from 8E5 cells (carrying one integrated provirus per genome) combined with DNA from HIV-negative primary (305, 153, 76, 38, 19 HIV DNA copies and 100,000 HIV negative cell equivalents per well). The following primers were used to target HIV-DNA: 5′-GGTCTCTCTGGTTAGACCAGAT-3′ (forward, HXB2 455–476) and 5′-CTGCTAGAGATTTTCCACACTG-3′ (reverse, HXB2 614–635) [38]. The amplification reaction was carried out in quadruplicates with a primer concentration of 0.5 µM, 10 µL SsoFast^TM^ EvaGreen® Supermix and 200,000 genomes as template in 20 µL total volume on a Bio-Rad CFX96^TM^ Real-time PCR Platform. PCR conditions were as follows: A denaturation step at 95°C followed by 45 cycles of 10 seconds at 95°C and 25 seconds at 62°C. Subsequently a melt curve was obtained heating from 65°C to 95°C with a 0.5°C increment for 5 seconds. Cycle cut off was set at 41 cycles. Amplification products were verified by melting curve. Copy number of HIV DNA per 1×106 PBMCs was calculated from the real-time PCR results. From the HIV DNA standard curve we set a lower limit of quantification of 25 copies HIV DNA/106 PBMCs.

### Neutralizing antibodies

CpG ODNs have previously been suggested to induce polyclonal B cell proliferation [39]. The effects of CPG 7909 administration on the production of HIV-1 neutralizing antibodies were measured in HAART-naïve trial participants since serum traces of antiretrovirals hamper the validity of the neutralization assay in persons on HAART. The neutralization assay has been described in details elsewhere [40].

### Antibody response

Total serum IgG specific to the HIV envelope antigen was measured by ELISA. Briefly, polystyrene MaxiSorp™ microtitre plates (Nunc, Roskilde, Denmark) were coated with capture antibody (D7324, Aalto Bio Reagents, Ireland) ON at 4°C. The plates were washed 5 times with washing buffer (PBS 0.05%Tween-20). Following block with 5% low-fat milk in PBS wells were incubated with recombinant 0.5 µg/well gp120 (Aalto Bio Reagents, Ireland). Serum samples were added at 3000× dilutions and incubated for 2 hours at RT. Following extensive washing in PBS 0.05% Tween-20 mouse monoclonal anti-human IgG biotin-conjugated antibody (Sigma, Missouri, USA) was diluted (1∶1,000) and added to the plates. After 2 hours plates were washed and streptavidin-HRP (R&D Systems, Minneapolis, MN, USA) was added for 30 minutes. After washing, 100 µl of TMB-plus substrate (Kem-En-Tec Diagnostics, Copenhagen, Denmark) was added. The reaction was stopped with 1.2 M H_2_SO_4_. Optical density was detected spectrophotometrically at 450–610 nm using a FLUOstar Omega microplate reader (BMG Labtech, Germany). Antibody concentrations were calculated using a reference obtained by pooling serum from 20 viremic patients producing a standard curve by a two-fold dilution series.

### T cell phenotype

Cryopreserved PBMCs were rapidly thawed and transferred in to 13 ml cold phosphate buffered saline solution (PBS). After wash 10^6^ cells were resuspended in FACS tubes with a staining buffer containing bovine serum albumin and incubated in the dark for 20 min with the following antibodies: α-CD3 conjugated PE-Cy7(BD Biosciences):, α-CD4 conjugated APC-H7(BD Biosciences):, α-CD27 conjugated APC(BD Biosciences):, α-CD38 conjugated PerCP-Cy5.5(BioLegend), α-CD45RA conjugated FITC(BioLegend) and α-CCR7 conjugated PE(BD Biosciences):. Samples were washed with 2 ml FACS flow buffer (BD Biosciences) and analyzed on a FACS Canto 2 flow cytometer (BD Biosciences). CD4+ subsets were defined as Naïve (T_N_: CD45RA+ CCR7+ CD27+), Central Memory (T_CM_: CD45RA− CCR7+/− CD27+), Effector Memory (T_EM_: CD45RA− CCR7− CD27−) and Terminally Differentiated (T_TD_: CD45RA+ CCR7− CD27−).

The CD3+CD4− population was used as an approximation for CD8+ cells. CD8+ subsets were defined as Naïve (CD45RA+ CD27+), Memory (CD45RA− CD27+) and Effector (CD45RA+/− CD27−). Data were analyzed using FlowJo v.7.6.5 (Tree Star, Ashland, OR).

### Antigen specific T cell stimulation

Cryopreserved PMBCs were rapidly thawed and subsequently rested over night at 37 °C, 5% CO_2_ in complete medium (RPMI 1640 supplemented with 1% FBS, 1% L-glutamine and 1% PenStrep) in 96-well round bottom plates (200.000 cells/150 µl/well). The following morning costimulatory antibodies (αCD28 and αCD49d; each at 1 µg/ml BD Biosciences), GolgiStop (0.7 µl/ml BD Biosciences), GolgiPlug (1 µl/ml BD Biosciences) and α-CD107 antibody APC (BD Biosciences) were added. PBMCs were incubated at 37 °C, 5% CO_2_ for 5 hours with overlapping 15-mer peptide pools encompassing HIV-1 Group M consensus Gag (obtained through the AIDS Research and Reference Reagent Program, Division of AIDS, NIAID, NIH). Each individual peptide in the pool was at a final concentration of 30 ng/ml. Complete medium was used for a negative control. Staphylococcal enterotoxin B (SEB) (final concentration 2 µg/ml Sigma-Aldrich) was used as a positive control.

At the end of 5 hours, cells were transferred to FACS tubes (10^6^ cells/tube), washed with PBS and stained with LIVE/DEAD Fixable Near-IR Dead Cell Stain Kit (Invitrogen, Denmark) for 30 minutes on ice. Cells were washed and α-CD4 conjugated PE-Cy7(BD Biosciences) and α-CD8 conjugated PerCP-Cy5.5(BD Biosciences) were added to stain for surface markers for 20 minutes on ice in the dark. Following a thorough wash, cells were fixed and permeabilized using the Cytofix/Cytoperm kit (BD Biosciences). α-IFN-γ conjugated FITC (BD Biosciences) and α-MIP-1β conjugated PE (BD Biosciences) was added for 20 minutes in the dark at RT. After a final wash the samples were analyzed on a FACS Canto 2 flow cytometer (BD Biosciences). Data were analyzed using FlowJo v.7.6.5 (Tree Star, Ashland, OR). For each patient, cytokine expression was determined by subtracting the background (the unstimulated control sample) from the peptide-stimulated sample. Cytokine expression is expressed as frequency of CD4+ or CD8+ cells. Only patients with samples available from both pre and post the 3^rd^ immunization were included in the analyses. Patients with less than 10,000 events at either time point were excluded from the analysis.

### Statistical analysis

Statistical analyses and graphical presentations were performed using GraphPad Prism, version 5.0 d (GraphPad software) and Stata Statistical Software, Release 12 (StataCorp). Wilcoxon signed rank test was used to analyze changes within the groups and Mann-Whitney test was used to compare groups. For Gaussian distributed data we used student's t test. Pearson correlation was used to evaluate proviral load change and CD8+ T cell immunity. P values below 0.05 were considered significant.

## Results

### Study population

**Figure 1:**
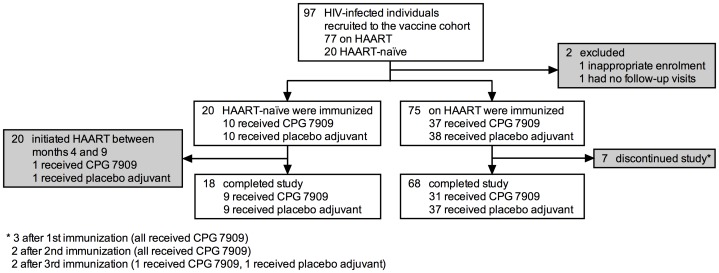
Disposition of study participants. HAART, highly active antiretroviral therapy.

Twenty HAART-naïve and 75 HAART-treated persons were included in the vaccine study. Nine participants did not complete the three dose vaccination series (figure 1). The placebo and CPG groups were similar in immune status and baseline characteristics at the time of inclusion (table 1).

**Table 1:** Baseline Characteristics of the Study Population at Time of Inclusion in the Study.

Characteristic	Control group (n = 26)	CpG 7909 group (n = 28)	P value
Sex			0.24
Male	21 (80.8)	26 (92.9)	
Female	5 (19.2)	2 (7.14)	
Race			0.23
White	24 (92.3)	28 (100)	
Other	2 (7.69)	0 (0.00)	
Age, median years (IQR)	48.9 (44.5–60.6)	49.5 (44.5–61.2)	0.58
Median BMI (IQR)	23.2 (21.9–24.8)	24.0 (22.0–25.3)	0.51
History of AIDS-defining event	7 (26.9)	9 (32.1)	0.77
CD4+ cell count, median cells/ µL (IQR)	617 (519–854)	758 (490–868)	0.80
Nadir CD4+ cell count, median cells/ µL (IQR)	215 768–260)	175 (50–243)	0.35
HIV RNA level, median log10 copies/mL	1.60	1.60	0.98
Duration of HAART, median years (IQR)	6.63 (2.91–10.8)	9.29 (6.74–10.7)	0.28
Quantifiable HIV DNA at 0 months (before 1st immunization)	22 (84.6)	21 (75.0)	0.51
Quantifiable HIV DNA at 3 months (before 2nd immuization)	24 (92.3)	23 (82.1)	0.42
Quantifiable HIV DNA at 9 months (before 3rd immunization)	21 (80.8)	22 (78.6)	1.00

Note: Data are no. (%) of patients, unless otherwise is indicated. BMI, body mass index; PBMCs, IQR, interquartile range.

### Impact of CPG 7909 on the proviral reservoir

To determine the potential impact of CPG 7909 on the viral reservoir, we focused on pre- and post-immunization proviral DNA levels among participants on suppressive HAART. Relative changes in proviral DNA were calculated before and after the 1^st^ immunization (at 0 and 3 months), before and after the 2^nd^ immunization (at 3 and 4 months), and before and after the 3^rd^ immunization (at 9 and 10 months). In each analysis, we excluded patients who had no available sample at one or both time points or had unquantifiable HIV DNA at the time of immunization (defined as <25 copies HIV DNA/10^6^ PBMCs). Twenty-one of the 75 patients included in the study were excluded from all three analyses. The remaining 54 patients were used in one or more of the analyses (table 1).

**Figure 2:**
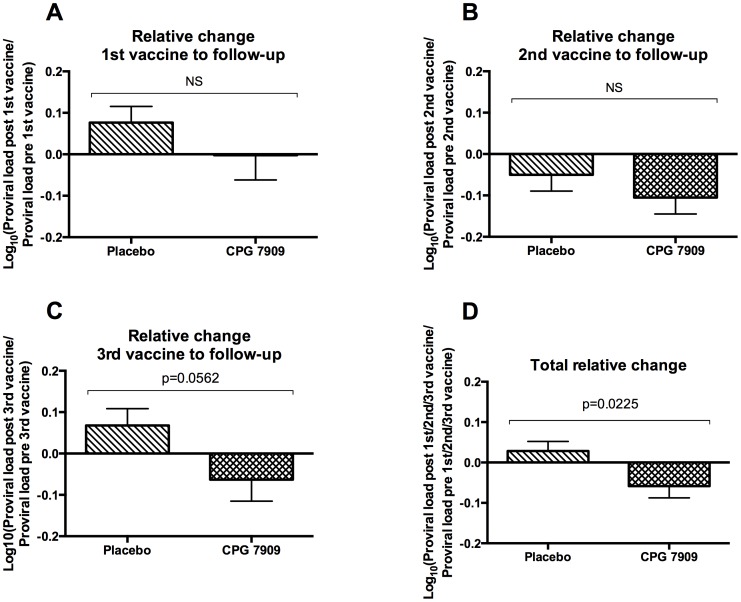
Proviral load. Relative changes in proviral load before and after immunization. **(A)** Before and 3 months after the 1^st^ immunization. N = 43 (placebo = 22, CPG = 21) **(B)** Before and 1 month after the 2^nd^ immunization. N = 47 (placebo = 24, CPG = 23) **(C)** Before and 1 month after the 3^rd^ immunization. N = 43 (placebo = 21, CPG = 22) **(D)** Pooled data from before and after all three immunizations. N = 133 (placebo = 67, CPG = 66). Bars show mean with SEM.

There were differences in effect following the 3 vaccine administrations. Following administration of the 1^st^ immunization proviral load in the CPG group was unchanged (mean change −0.47% (95% CI: −25.3–32.5) while there was a mean increase in proviral load in the placebo group of 19.3% (95% CI: −1.00–43.7), p = 0.27 (figure 2A). Following the 2^nd^ immunization there was a minor decrease in the placebo group of −10.9% (95% CI: −26.2–7.40) not significantly different from zero and a larger decrease in the CPG group: −21.5% (95% CI: −35.1–−5.18), p = 0.33 (figure 2B). After the 3^rd^ immunization we again observed a mean increase in the placebo group of 16.9% (95% CI: −3.77–42.0) and a decrease in the CPG group of −13.5% (95% CI: −32.7–11.1), p = 0.056. (figure 2C). When pooling the data from before and after all three immunizations we observed a mean reduction in proviral DNA in the CPG group of 12.6% (95% CI: −23.6–0.0) following each immunization whereas proviral DNA in the placebo adjuvant group remained largely unchanged (6.7% increase; 95% CI: −4.2–19.0 after each immunization, p = 0.02) (figure 2D).

For actual HIV-DNA levels at each time point and proviral load changes over the full follow-up period see figures S1 and S2.

### CPG 7909 did not increase production of HIV-specific antibodies

**Figure 3:**
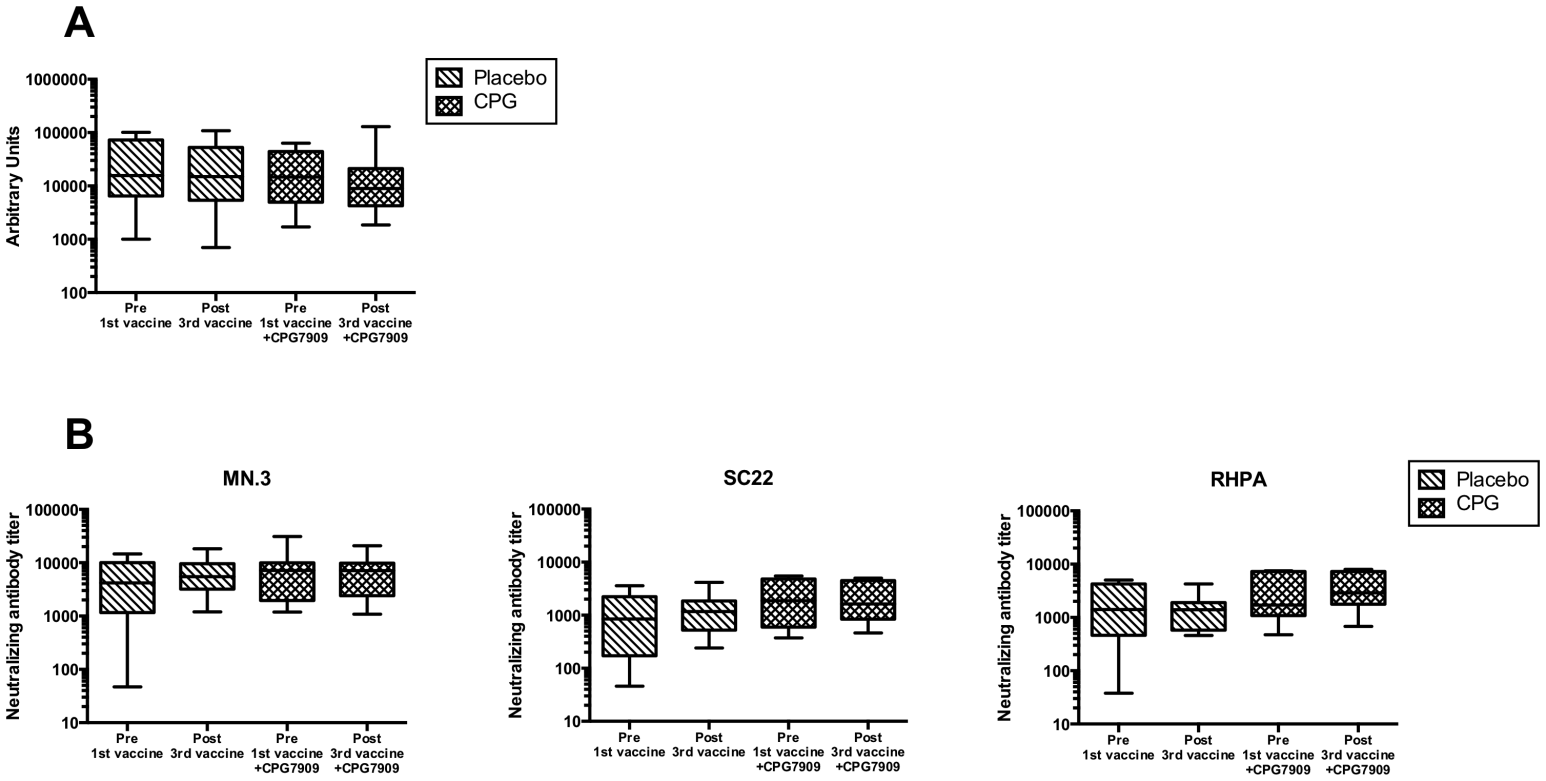
HIV-specific antibodies. **(A)** Quantitative antibodies **(B)** Neutralizing antibodies.

There was no change in quantity of HIV envelope antibodies in serum in either of the two arms during the course of the study from baseline to 10 months (Figure 3A). Furthermore, comparing the neutralizing capacities of serum antibodies against three different HIV envelopes (in the 18 HAART-naïve trial participants) we found no effect of CPG 7909 on the neutralizing capacity of antibodies against these strains (Figure 3B).

### CPG 7909 administration did not induce T cell memory phenotypic shifts

**Figure 4:**
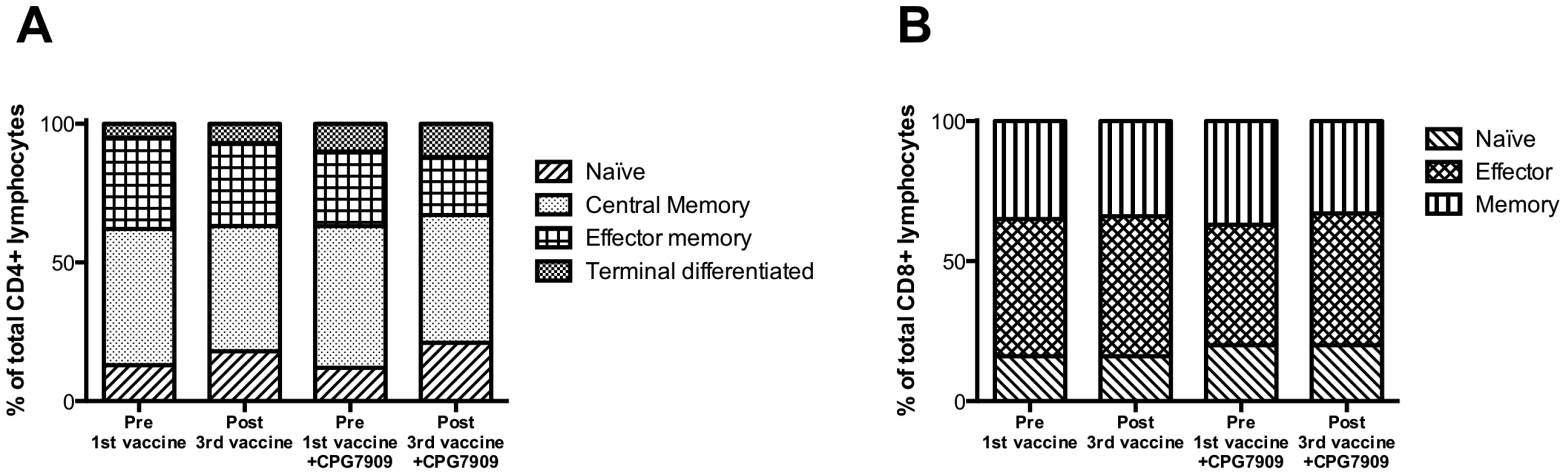
T-cell phenotype at baseline and the end of the study in (A) the CD4+ T cell and (B) CD8+ T cell compartment.

We characterized the phenotypes of CD4+ and CD8+ T cells at baseline and 10 months to determine whether changes in HIV proviral DNA could be attributed to changes in T cell subsets. There were discrete fluctuations in the distribution of CD4+ T cell memory subsets from baseline to the end of the study, but these changes were similar in the two study groups. The same applied to the distribution of CD8+ T cell subsets (figure 4).

There was no correlation between change in HIV DNA and change in the proportion of memory CD4+ T cell over the full course of the study (Figure S3).

### Effect of CPG on HIV-specific T cell immunity

**Figure 5:**
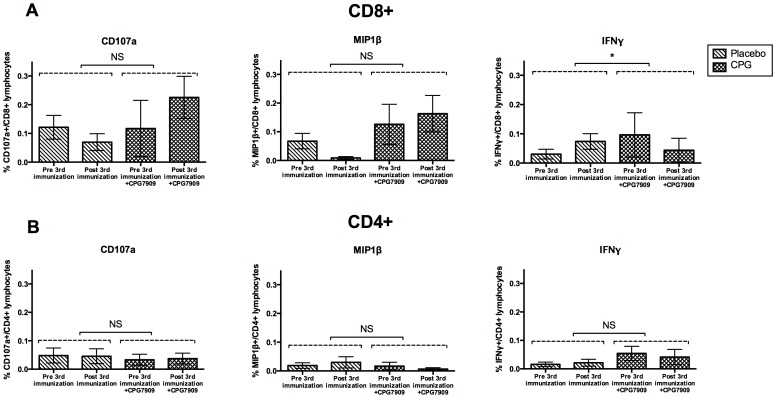
HIV-specific T cell immunity. Percentage of cells expressing CD107a, MIP1β and IFNγ before and after the 3^rd^ immunization in the **(A)** CD4+ T cell compartment and **(B)** CD8+ T cell compartment. Bars show mean with SEM. N = 17 (placebo = 10, CPG = 7). Statistical comparisons were made between the change from before and after the 3^rd^ immunization in the two groups.

The proportion of HIV-specific CD8+ cells expressing the degranulation marker CD107a increased following the 3^rd^ vaccine in the CPG group whereas it decreased in the placebo group (p = 0.13). The same was apparent regarding the expression of macrophage inflammatory protein 1β (MIP1β) in CD8+ cells, where we observed a decrease in the placebo group and an increase in the CPG group after the 3^rd^ vaccine (p = 0.09). The proportion of CD8+ cells expressing IFNγ increased in the placebo group, while it decreased in the CPG group (p = 0.0005) (figure 5A).

In contrast, the findings in the CD4+ compartment revealed no changes in either CD107a, MIP-1 β or IFN-γ expression in either the placebo group or the CPG group (figure 5B). The positive proportion of each functional marker was low, thus excluding analyses of poly-functionality.

### HIV-specific CD8+ response and proviral load change

**Figure 6:**
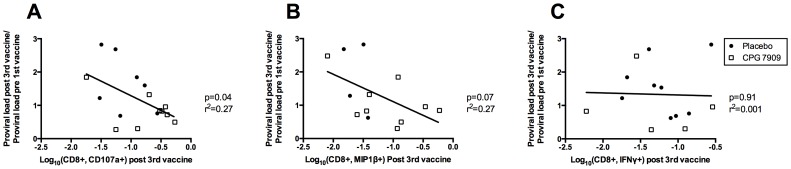
HIV-specific CD8+ response and proviral load change. **(A)** CD107a expressing CD8+ T cells (n = 16, placebo = 7, CPG = 9) **(B)** MIP1β expressing CD8+ T cells (n = 13, placebo = 4, CPG = 9) and **(C)** IFNγ expressing CD8+ T cells (n = 14, placebo = 9, CPG = 5) correlated with the relative change in proviral load from before the 1^st^ immunization to 1 month after receiving the 3^rd^ immunization.

To investigate associations between immune response at the end of follow-up and change in proviral load, we correlated the expression of CD107a, MIP1β and IFNγ in CD8+ T cells 1 month after receiving the 3^rd^ vaccine with the relative change in proviral load before the 1^st^ vaccine and 1 month after the 3^rd^ vaccine (Figure 6A–C). For MIP1β we found a correlation (p = 0.04) between decreasing proviral load and expression of MIP1β in HIV-specific CD8+ T cells (Figure 6B). A similar association was observed between decreasing proviral load and expression of CD107a in HIV-specific CD8+ T cells though not statistically significant (p = 0.07). However, there was no association between IFNγ and change in proviral load (p = 0.91) (Figure 6C).

## Discussion

In this selected group of virologically suppressed patients with quantifiable HIV DNA, low dose TLR9 agonist (CPG 7909) in combination with pneumococcal vaccines reduced the proviral reservoir in peripheral blood as compared with pneumococcal vaccine alone. Changes in proviral DNA occurred simultaneously with alterations in the proportions of HIV-specific CD8+ T cells. In particular, we found that the expression of HIV-specific MIP1β in CD8+ T cells correlated with a decrease in proviral load. These findings may indicate that TLR9 agonist administration cause reactivation of integrated HIV DNA and boosting of HIV-specific cytotoxic CD8+ T cell immunity *in vivo*. To our knowledge this is the first *in vivo* study to investigate the effect of TLR9 agonists on the proviral HIV reservoir and despite the limitations inherent to this post-hoc analysis, our results provide the rationale for further studying the ability of TLR-agonists to modulate immune function and disrupt proviral latency.

*In vitro* studies have suggested that TLR9 agonists could induce HIV production in latently infected cells providing a possible therapeutic mechanism to disrupt HIV latency [31], [32]. However, as we were unable to assess changes in residual viremia or viral transcription, it remains uncertain whether the decrease in proviral reservoir observed in this study can be attributed to induced virus production in latently infected cells, effects on HIV-specific T cell immunity, or non-specific effect on other immune effector cells, or a combination of the three. It has previously been shown that besides increasing antibody production, CPG 7909-adjuvanted pneumococcal vaccination also enhances antibody-independent cellular immunity [29]. In addition to B cells, TLR9 is also abundant in plasmacytoid dendritic cells (pDCs) [41]. pDCs possess increased ability to induce apoptosis of HIV-infected cells after CPG treatment [42] Furthermore pDCs hold antiviral properties and secrete IFN-α/β in response to viruses, which activates and enhances cytotoxicity of NK cells and CD8+ T cells [43]–[45] and thereby possibly promotes anti-HIV immunity. Though CpG ODNs are well-characterized B cell adjuvants and TLR9 stimulation via CpG ODNs have been suggested as a mechanism to maintain serological memory by polyclonal activation of human memory B cells[39], we observed no measurable effect on neither the quality or the quantity of HIV-specific antibodies. Also, we found no difference in the change of CD4+ and CD8+ T cell phenotypes between the two groups through the course of the trial. The absence of phenotype shift suggests that the observed decline in proviral load associated with CPG administration is owing to killing or apoptosis of the cells harbouring integrated HIV DNA and not homeostatic proliferative differences between T cell subsets in the two groups.

The strategy to eradicate HIV infection by reactivating HIV-1 expression in latently infected cells requires activation of viral transcription in latently infected cells followed by the removal of infected cells expressing viral products. A recent *in vitro* study suggested that reactivation of virus production in latently infected resting cells was insufficient to eradicate these cells. Only after stimulation of HIV-1 specific cytolytic T cells was efficient killing of latently infected cells achieved [19]. In a previous study a decline in replication-competent virus was observed in a group of HIV-infected individuals treated with intermittent IL-2 plus HAART [23]. In two of these patients, where replication-competent virus was undetectable during treatment, rebound viremia was observed within weeks after stopping HAART [46]. This shows that despite extremely low frequencies of resting cell infection, virological control is not sustained in the absence of HAART and enhancement of HIV-specific CTL function is likely needed to achieve a cure for HIV. A more recent observational study arrived at the same conclusion [38]. Data to support the importance of HIV-specific immunity was reported in an analysis of HIV reservoir decline following therapeutic vaccination in HAART-suppressed patients although the effect was transient. The observed change in reservoir size was negatively correlated with the change in IL-2 producing HIV-specific CD8+ T cells from baseline to 6 weeks after vaccination [21].

A rare subset of HIV-infected patients termed ‘elite controllers’ (EC) are able to spontaneously control the HIV-infection to a point where the viral loads are undetectable by standard clinical assay [47]. One mechanism to explain this control is enhanced cytotoxic function in EC compared with progressors. Studies have shown that CD8+ T cells from EC have increased ability to suppress replication of HIV in CD4+ T cells [48], [49]. This further emphasizes the need to enhance cellular immunity, in combination with attempts to reactivate viral transcription in latently infected cells.

We recognize several limitations in our study. As HIV DNA was not quantifiable in all study subjects, we had to further select for inclusion into the analyses presented here limiting the statistical power and introducing a selection bias. Patients with low proviral load are likely to maintain higher levels of HIV-specific immunity [50] and this group of patients would therefore be more likely to respond favorably to the effects of CpG ODNs on the proviral reservoir. Thus, their absence from our analyses could have biased our results towards underestimating the effect of CPG 7909 on the proviral reservoir. Moreover, the study was designed to compare the immunogenicity of pneumococcal vaccines adjuvanted with CPG 7909 and thus not intended to stringently evaluate the effect of CPG 7909 on the proviral reservoir. For this reason, CPG 7909 administration and sampling were spaced too far apart and was insufficient to fully explore early effects of CPG on virus activation per se and any low-level viremia. Previously, an increase in viral load was observed in HIV-infected individuals after 8 days continuous intravenous (IV) infusion of a HIV-gag antisense ODN containing a CPG motif. Levels of HIV RNA were increased by day 4 and even more by day 8, but returned to a level similar to the placebo group six days after treatment completion [35] indicating that to detect ‘blips’ in HIV RNA, sampling has to be placed within days following CPG administration. In our study follow-up was placed 3 months and 1 month after the 1^st^ and 2^nd^/3^rd^ vaccine respectively. Finally, additional exploratory immunological analyses of HIV-specific T cell immunity (e.g. perforin/granzyme expression on CD8+ T cells) could not be performed due to shortage of cryo-preserved PBMCs. However, earlier reports have concluded that the degranulation marker CD107a is a good correlate of the quality of the HIV-specific CD8+ T cell response [51].

In conclusion we report that TLR9-adjuvanted pneumococcal vaccination decreased proviral load in virologically suppressed HIV-infected individuals and these reductions in proviral load correlated with increasing levels of HIV specific CD8+ T cells. Our study suggests that CPG could impact proviral load by enhancing immune-mediated clearance of infected cells. Further investigation into the potential effect of TLR9 agonists on HIV latency is warranted.

## Supporting Information

Figure S1 **Relative change in proviral load from before the 1^st^ immunization to 1 month after receiving the 3^rd^ immunization.** N = 34 (placebo = 19, CPG = 15).
(TIFF)

Figure S2 **Proviral load at each time point in (A) the placebo group and (B) the CPG group.** Bars show median with interquartile range.
(TIFF)

Figure S3 **Proportion of memory CD4+ T cells and proviral load change.** Relative change in the proportion of memory CD4+ T cells out of the total CD4+ T cell population correlated with relative change in proviral load from before the 1^st^ immunization to 1 month after receiving the 3^rd^ immunization. **(A)** The placebo and CPG 7909 group combined. **(B)** Placebo group. **(C)** CPG 7909 group.
(TIFF)
