# Supplementary figures and images for: Administration of a Toll-Like Receptor 9 Agonist Decreases the Proviral Reservoir in Virologically Suppressed HIV-Infected Patients
Source: PLoS One. 2013 Apr 26;8(4):e62074. doi: 10.1371/journal.pone.0062074 (PMC3637371; doi:10.1371/journal.pone.0062074)

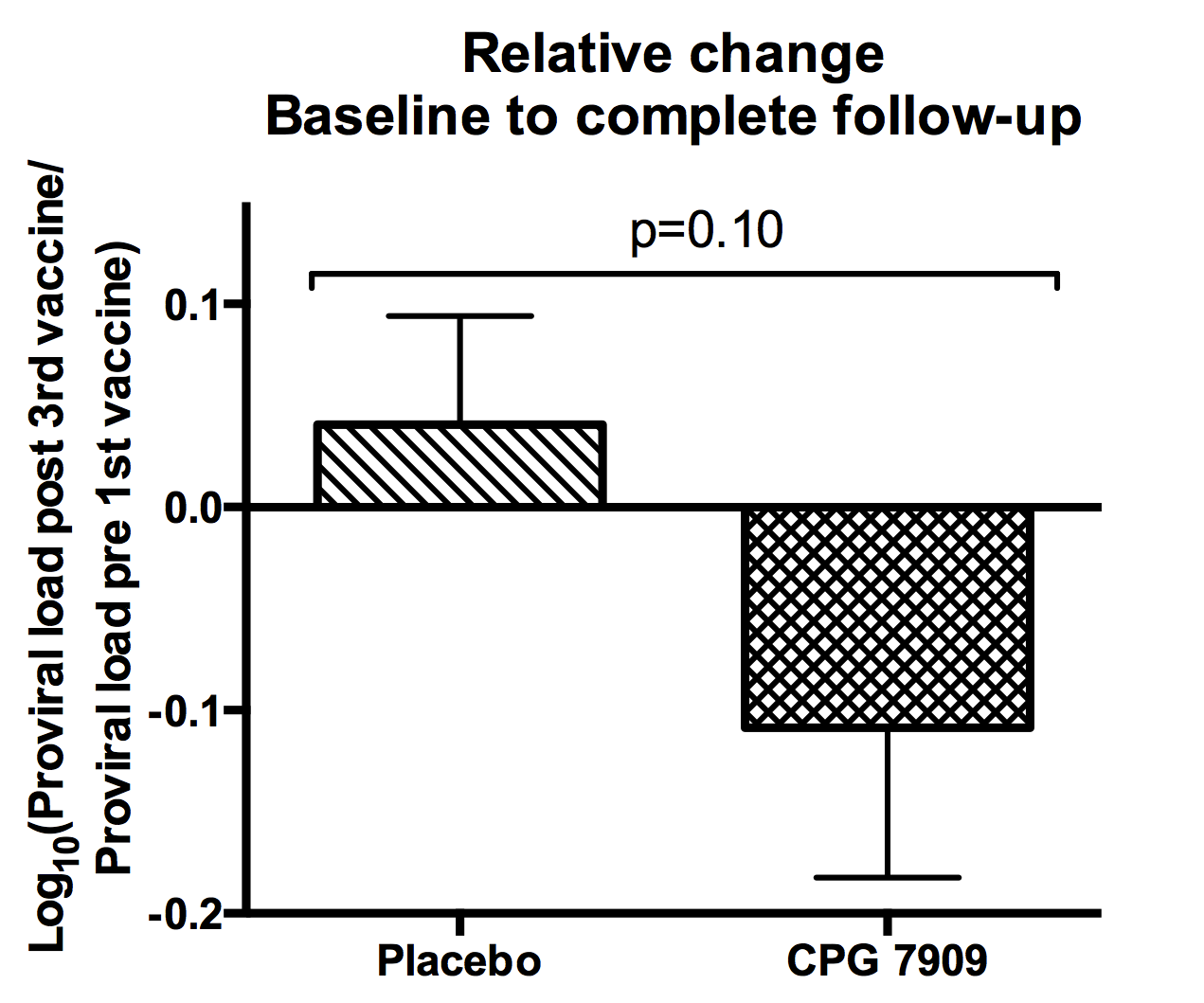

Supplement: Figure S1 — Relative change in proviral load from before the 1st immunization to 1 month after receiving the 3rd immunization. N = 34 (placebo = 19, CPG = 15). (TIFF) [file pone.0062074.s001.tiff]

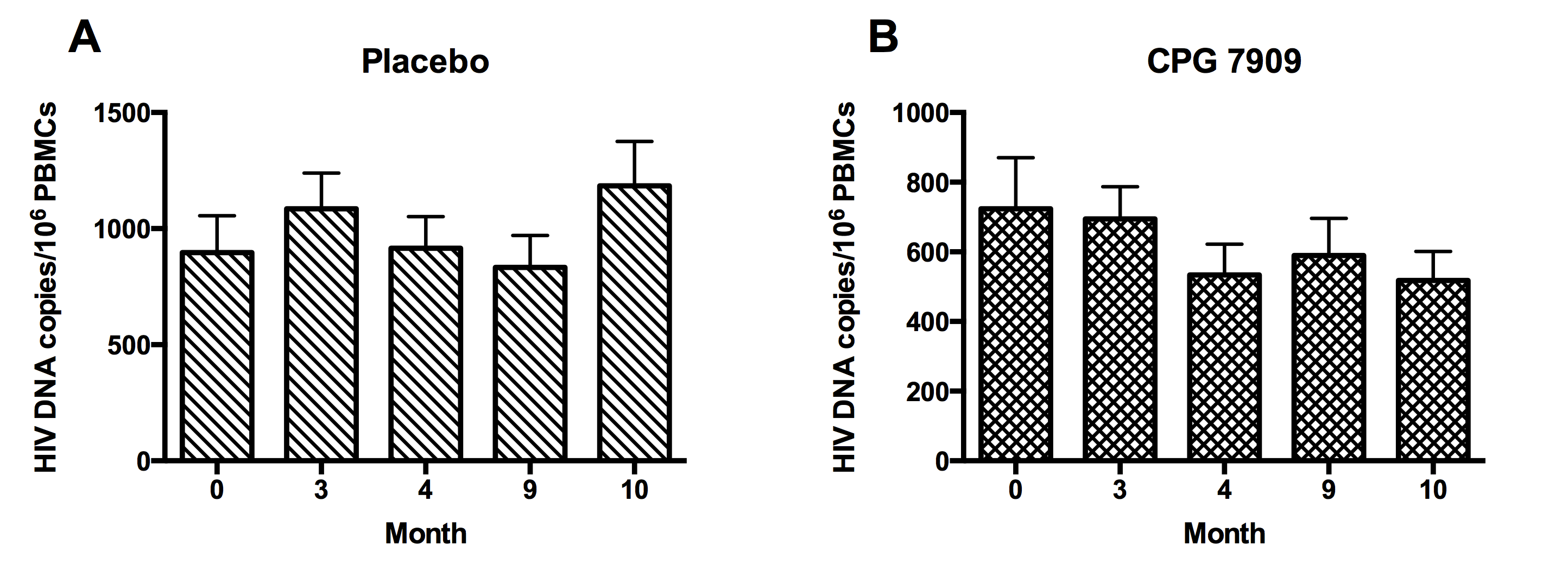

Supplement: Figure S2 — Proviral load at each time point in (A) the placebo group and (B) the CPG group. Bars show median with interquartile range. (TIFF) [file pone.0062074.s002.tiff]

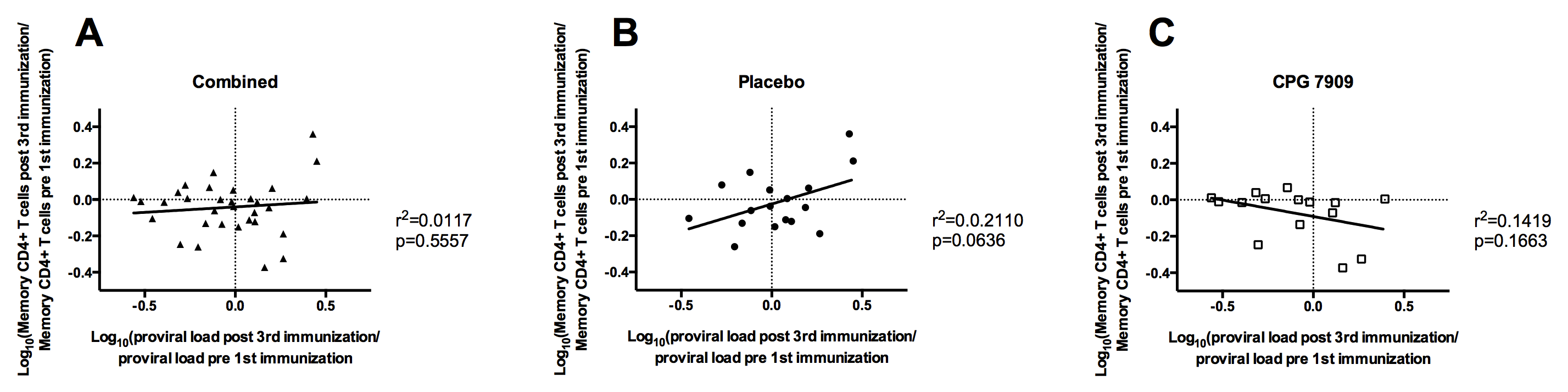

Supplement: Figure S3 — Proportion of memory CD4+ T cells and proviral load change. Relative change in the proportion of memory CD4+ T cells out of the total CD4+ T cell population correlated with relative change in proviral load from before the 1st immunization to 1 month after receiving the 3rd immunization. (A) The placebo and CPG 7909 group combined. (B) Placebo group. (C) CPG 7909 group. (TIFF) [file pone.0062074.s003.tiff]
